# Computational Identification of Novel Stage-Specific Biomarkers in Colorectal Cancer Progression

**DOI:** 10.1371/journal.pone.0156665

**Published:** 2016-05-31

**Authors:** Ashok Palaniappan, Karthick Ramar, Satish Ramalingam

**Affiliations:** Faculty of Allied Health Sciences, Chettinad Academy of Research and Education, Kelambakkam, Tamil Nadu 603103, India; University of Kansas Medical Center, UNITED STATES

## Abstract

It is well-known that the conversion of normal colon epithelium to adenoma and then to carcinoma stems from acquired molecular changes in the genome. The genetic basis of colorectal cancer has been elucidated to a certain extent, and much remains to be known about the identity of specific cancer genes that are associated with the advancement of colorectal cancer from one stage to the next. Here in this study we attempted to identify novel cancer genes that could underlie the stage-specific progression and metastasis of colorectal cancer. We conducted a stage-based meta-analysis of the voluminous tumor genome-sequencing data and mined using multiple approaches for novel genes driving the progression to stage-II, stage-III and stage-IV colorectal cancer. The consensus of these driver genes seeded the construction of stage-specific networks, which were then analyzed for the centrality of genes, clustering of subnetworks, and enrichment of gene-ontology processes. Our study identified three novel driver genes as hubs for stage-II progression: *DYNC1H1*, *GRIN2A*, *GRM1*. Four novel driver genes were identified as hubs for stage-III progression: *IGF1R*, *CPS1*, *SPTA1*, *DSP*. Three novel driver genes were identified as hubs for stage-IV progression: *GSK3B*, *GGT1*, *EIF2B5*. We also identified several non-driver genes that appeared to underscore the progression of colorectal cancer. Our study yielded potential diagnostic biomarkers for colorectal cancer as well as novel stage-specific drug targets for rational intervention. Our methodology is extendable to the analysis of other types of cancer to fill the gaps in our knowledge.

## Introduction

Colorectal cancer is the third most common cancer in men, and the second in women worldwide according to International Agency for Research on Cancer, World Health Organization [1]. In recent years, the incidence of colorectal cancer has significantly increased in newly developed countries where the risk was once low. Despite the existence of screening and preventive strategies, colorectal cancer remains a major public health problem. Cancer mortality is significantly correlated with the stage of development of the cancer and could be reduced if cases are detected and treated early. The 5-year survival rate of colorectal cancer patients declines significantly with the stage of the cancer. Most importantly, the 5-year survival rate for patients with distant metastases is only 10%. Considering the fact that metastases are the cause of 90% of cancer deaths[2], there are no effective drugs available to curtail the process of cancer spreading to different organ systems. Treatment of metastases remains a major challenge, not least because our knowledge of factors responsible for cancer progression and metastases is far from complete. With an increased trend in incidence and death rates of colon cancer, and the unpredictability of factors responsible for the metastatic potential of the localized tumor, we prioritized our efforts towards identifying the genes responsible for colon cancer progression. Increasing our current knowledge of the genes and pathways that play important roles in the progression of cancer from one stage to the next could prove very useful in early-stage diagnosis as well as identifying targets for personalized cancer therapy.

**Fig 1:**
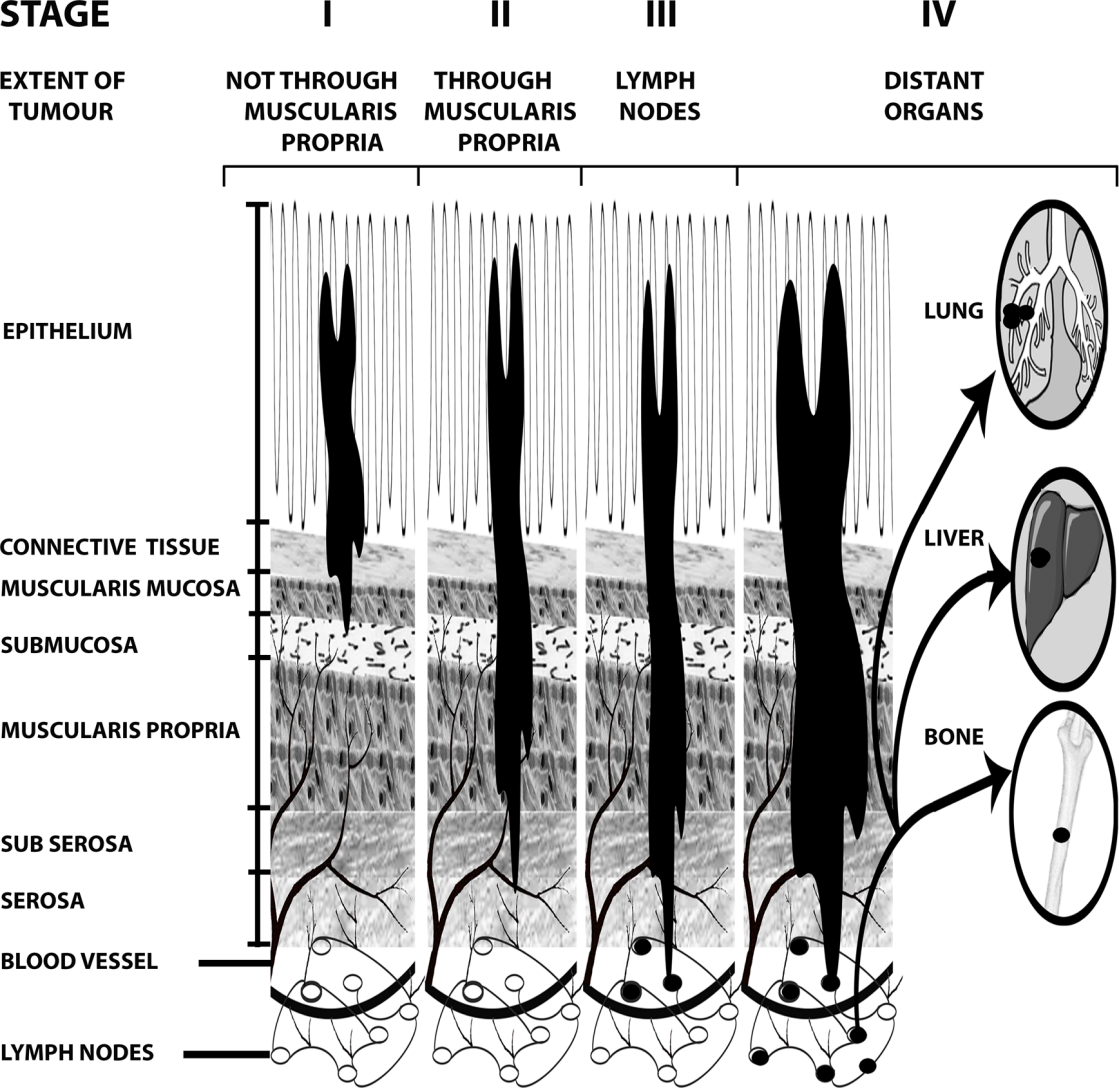
Staging of colon cancer. The American Joint Committee on Cancer (AJCC) has staged the colorectal cancer based on the anatomical extent of the disease. Stage I: Tumor that is limited to the mucosal layer (T1) or muscularis propria (T2), without involvement of any lymph node or distant metastatic organs. Stage II: Tumor that penetrates the muscularis propria (T3) or invades nearby organs or structures (T4), without involvement of any lymph node or distant metastatic organs. Stage III: Tumor stages with lymph node metastasis but without distant metastasis. Stage IV: Any tumor stage and lymph node status with distant organ metastasis.

We have earlier published on important roles of two genes on colon cancer, *CELF2* a putative tumor suppressor gene [3,4] and *RBM3* a proto-oncogene [5]. Here we have attempted to identify more novel and key genes underpinning colon cancer progression using the available data from the TCGA consortium [6]. Mutations in colon cancer are complex and unclear due to the presence of passenger and driver genes even within the same tumor. Much effort has focused towards identifying driver genes. The aim of the current study is to utilize methods of network analysis to identify novel biomarkers responsible for the colorectal cancer progression to each stage. The differential anatomical penetration of the cancer for each stage is shown in Fig 1.

## Materials and Methods

### Dataset

TCGA datasets annotated by the stage of cancer were retrieved from the DriverDB [7] by performing the following meta-analysis. We selected colon adenocarcinoma as the tissue of interest, and specified ‘tumor stage’ as the clinical criteria. We obtained datasets for each stage of colon adenocarcinoma, namely stage I, stage II, stage III, and stage IV of colon adenocarcinoma.

### Identification of consensus driver genes

Framing the stage of tumor as the unit of analysis, we used the following tools to identify driver genes: ActiveDriver[8], Dendrix[9], MDPFinder[10], Simon[11], Netbox[12], OncodriveFM[13], MutSigCV [14], and MEMo [15]. To obtain the consensus driver genes, we determined the overlap between the predictions of the various tools for a given stage. The selective advantage conferred by driver genes to the growth of tumor cells could be either gain of function or loss of function events (for e.g., oncogenes are gain-of-function and insensitivity to tumor-suppressor is a loss of function). We filtered for driver genes that were identified by at least three tools and obtained the consensus prediction of driver genes for each stage.

### Novel driver genes

To identify novel driver genes, we subtracted the driver genes of stage I from the driver genes of stage II to ensure stage II-specific driver genes in the progression of cancer. In a similar manner, we obtained stage III-specific and stage IV-specific driver genes. To eliminate non-specific driver genes from the analysis, we screened each stage against a background of driver genes obtained from pooling all samples of colon adenocarcinoma regardless of stage of cancer. This set of non-redundant stage-specific driver genes was further screened against the Cancer Gene Census v68[16] to filter out any remaining known cancer genes. Thus we obtained novel and stage-specific driver gene sets for further analysis.

### Network analysis

**Fig 2:**
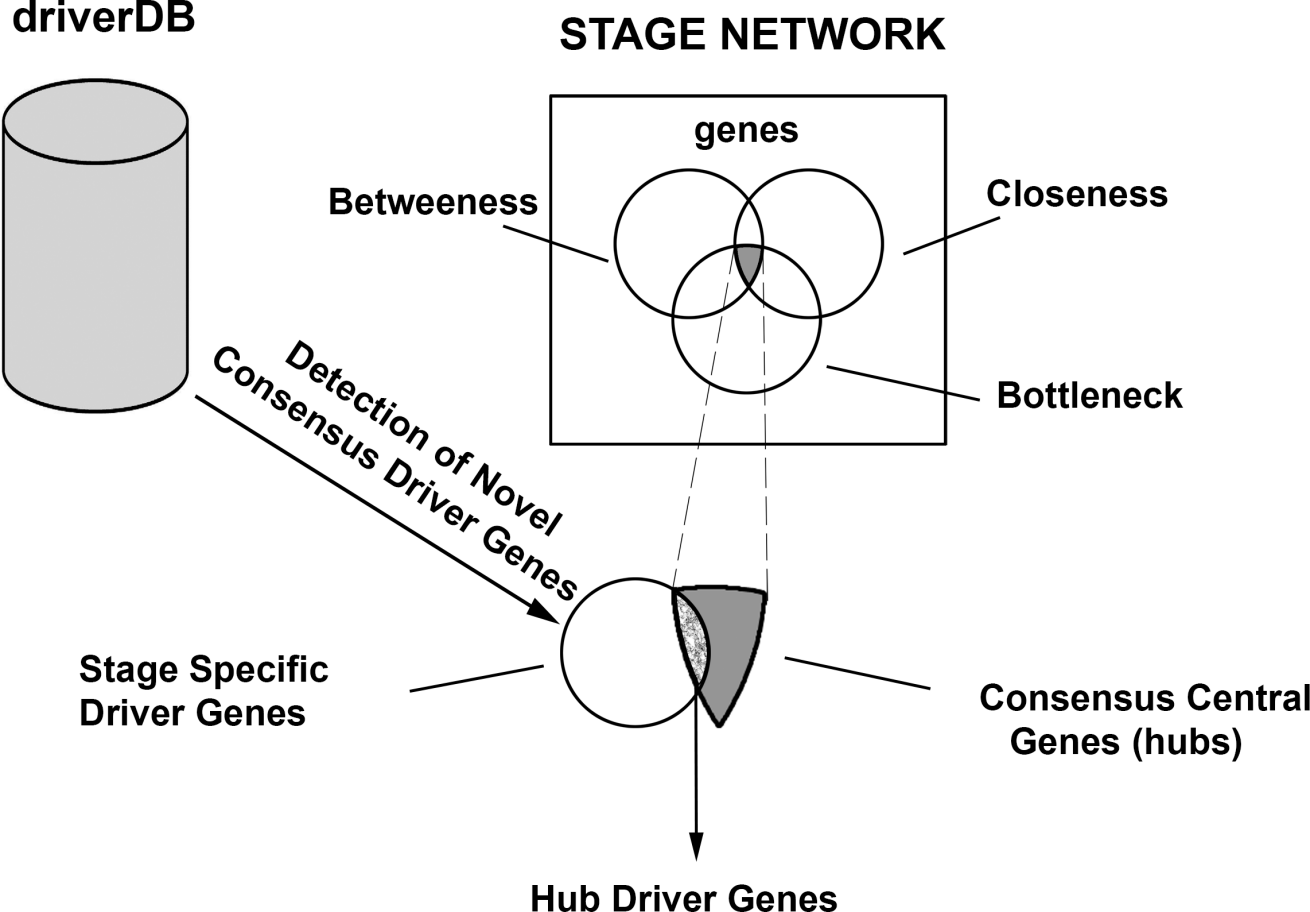
Identification of hub driver genes. Consensus novel driver genes were identified for each stage from driverDB data. Consensus central genes (‘hubs’) were identified from each stage-specific network. The overlap between these two sets of genes yields ‘hub driver’ genes for each stage.

The construction and analysis of stagewise networks were aided by Cytoscape[17]. The driver gene sets identified above were used to seed the construction of the corresponding stage-specific network using the Genemania tool [18]. We searched for the following types of interactions of the stage driver genes: ‘physical’, ‘protein-protein interactions’ and ‘predicted’. This yielded stage-wise networks. To analyze the topological properties of each network, we used NetworkAnalyzer[19]. The degree distribution of each network was calculated and the goodness of fit with a power-law distribution was determined using the coefficient of determination (R^2^). A high R^2^ implied the existence of fat tails in the degree distribution, indicating that some genes played the role of hubs. Alteration of function of these genes due to mutation, translocation or copy number variation could result in deleterious genes damaging cellular activity. To analyze the structure of the stage-wise networks, we performed centrality analysis, modularity analysis and Gene Ontology analysis. Centrality analysis identified the central nodes in each stage-specific network by various metrics using Centiscape [20]. Three metrics of centrality were used to rank the genes, viz. the between-ness centrality, closeness centrality and bottleneck centrality. These metrics were chosen for their measurement of complementary properties of node importance. The top 15 genes from each measure were chosen, and their intersection was determined to yield a consensus set of central genes for each stage (Fig 2). These are the “hub” genes identified in our work and discussed individually below. This stage-specific consensus set was compared with the set of driver genes for each stage. A gene common to both sets is a driver and a hub. Such genes are termed ‘hub’ driver genes for each stage. We then analyzed the clustering pattern of the stage-wise networks using the ModuLand algorithm [21]. The clusters obtained are indicative of driver sub-networks for the stage-wise progression of cancer. Finally, we interrogated each of these networks for Gene Ontology (GO) enrichment using BiNGO [22]for three GO terms: biological process, molecular function and cellular component. To correct for the false discovery rate with p-values in multiple hypothesis testing, we applied the Benjamini-Hochberg filter in BiNGO and obtained the q-values[23].

## Results and Discussion

Table 1 shows the number of driver genes at each step of our screening procedure. The final set of novel driver genes for each stage used in the subsequent Genemania search is shown in S1 Table. We analyzed the degree distribution of each of the stage wise networks and found that the node distribution of all the three networks conformed better to a power law distribution than a linear model (S2 Table). A power-law fit implies the presence of a few highly connected nodes (i.e. hubs)in the network. In general, hubs could predispose vulnerability to disease. Mutations in hub genes could lead to functional alterations in the corresponding protein which could lead to changes in its interactions with other proteins. This could lead to a cascading failure in the network and cause disease [24]. In this context, a power law behavior implies that mutations in hub genes could increase susceptibility to the hallmarks of cancer[25]and facilitate the spread of the perturbation in the network. Therefore, identification of the hub nodes could pinpoint the key genes whose failure would underscore the progression of cancer. Though our method of network construction was blind to the number of components, all our resultant network models were single connected giant components (S3 Table).

**Table 1:** From tumor genome-sequencing data to network reconstruction.

Stage	No. of Samples	Consensus driver genes	Subtraction of previous stage	Screening Against background	Screening against Cancer Gene Census	Network reconstruction #node; #edge
II	108	52	47	34	27	109; 396
III	75	56	43	35	31	109; 199
IV	28	49	43	32	30	115; 297

The number of driver genes at each step leading to the network reconstruction.

### Analysis of progression to stage II

**Fig 3:**
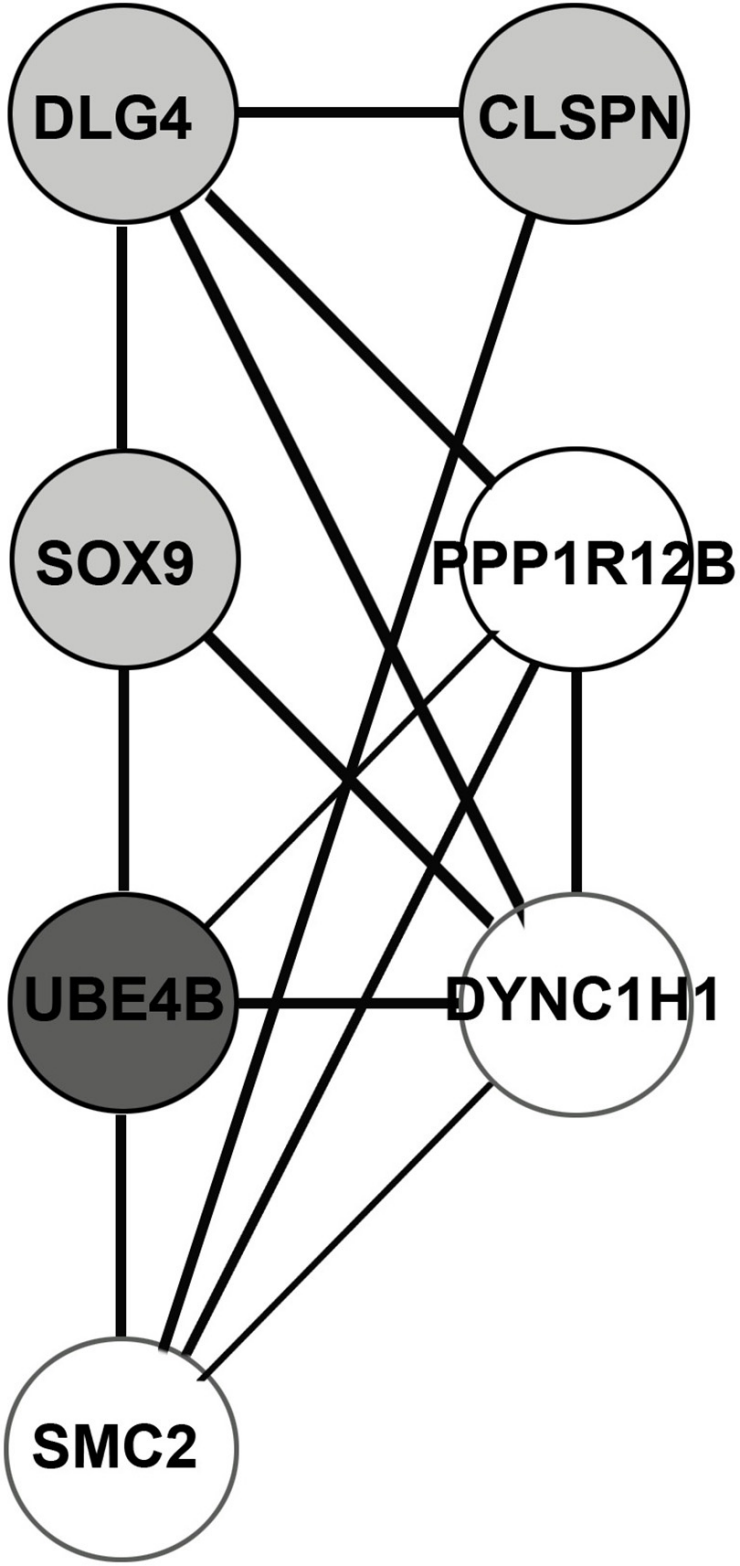
Network of cluster centres of stage-II network. Clustering was done using Moduland.

The reconstructed stage-II specific network (with node and edge attributes) is given in S1 File. Table 2 shows the central genes identified by centrality analysis for the stage II network. For progression to stage II, three hub driver genes were identified: DYNC1H1, GRIN2A, GRM1. Eight non-driver hub genes were identified: DLG4, SMC2, PLCG1, GRIN2B, RHOG, SMC1A, GRIN1, CAMK2A. Table 3 shows the modules in the network obtained by ModuLand analysis. Seven significant modules were obtained each centered at a different gene (Fig 3). Each module could function as a driver subnetwork for the progression of cancer to stage II. The most interesting module was that centered at DLG4, which also had the driver genes GRIN2A, GRM1, LRRC7 as its members. Of these, GRIN2A and GRM1 are also central genes. A gene ontology analysis for biological process yielded the key pathways in which these central driver genes played a role. The glutamate signaling pathway was identified as a key enriched biological process (q-value < 0.001). GRIN1, GRIN2A and GRIN2B emerged as key hub genes that could modulate this pathway. Among molecular functions, the glutamate receptor activity emerged significant (q-value < 0.001) in which GRM1 was implicated. The cellular component ontology was enriched in ionotropic glutamate receptor complex and the synapse (q-values < 0.001) (Table 4).

**Table 2:** Centrality analysis of stage-II network.

S.No.	Betweenness centrality	Closeness centrality	Bottleneck centrality	Consensus Centrality (hubs)	Hub+driver genes
1	DLG4	DLG4	DLG4	DLG4	DYNC1H1
2	SMC2	GRIN2B	DYNC1H1	SMC2	GRIN2A
3	DYNC1H1	GRIN2A	SMC2	DYNC1H1	GRM1
4	PLCG1	GRIN1	PLCG1	PLCG1	
5	GRIN2B	CAMK2A	CAMK2A	GRIN2B	
6	GRIN2A	PLCG1	RHOG	GRIN2A	
7	RHOG	DYNC1H1	NF2	RHOG	
8	SMC1A	RHOG	SMC1A	SMC1A	
9	GRM1	LRRC7	GRIN2A	GRM1	
10	NF2	GRIA1	GRM1	GRIN1	
11	GRIN1	GRM1	HRSP12	CAMK2A	
12	NCAPH	DLG2	GRIN2B		
13	CAMK2A	DYNLL1	LRRC7		
14	SOX9	SMC2	RAD21		
15	CHEK1	SMC1A	GRIN1		

The top 15 genes obtained by applying each centrality metric are given. The genes at the intersection of all the three metrics are designated as ‘hubs’. Genes that are both hub and driver are shown.

**Table 3:** Moduland decomposition of stage-II network.

S. No	Module centre	Eff. size of module	Driver gene members	Hub gene members
1	DLG4	43	GRIN2A, GRM1, LRRC7	CAMK2A, DLG4, GRIN1. GRIN2A, GRIN2B, GRM1, PLCG1, RHOG
2	SMC2	17	NCAPD2, STAG1	SMC1A, SMC2
3	DYNC1H1	6	DYNC1H1	DYNC1H1
4	PPP1R12B	9	PPP1R12B, KCNQ5	
5	UBE4B	6	UBE4B	
6	CLSPN	4	CLSPN	
7	SOX9	5	SOX9	

Each module is represented by its centre and effective size. Hub genes identified by centrality analysis and driver genes are indicated by their module membership. Modules with the maximum number of hub genes could function as driver subnetworks in the pathogenesis of disease progression.

**Table 4:** GO enrichment analysis of Stage-II network.

GO- ID	q-value	%size > 20	Description	Hub genes	Other genes in the network
7059	3.10E-12	N	chromosome segregation	SMC2, SMC1A	PDS5B, SMC3,SMC4,NCAPD2,NCAPH,RAD21, NDEL1, NCAPG, STAG2,NEK6,STAG1
7215	1.62E-10	Y	glutamate signaling pathway	GRIN1,GRIN2A,GRIN2B	HOMER3, GRIN3B, GRIA4,HOMER1, HOMER2
45202	1.62E-10	N	Synapse	GRIN1,GRIN2A,GRIN2B, GRM1, DLG4, CAMK2A	GABBR1,NLGN2,GRIN3B,GRIA4,CDH2,HOMER1,ADORA1,HOMER2,SLC17A7,SLC32A1,GRIA1,HOMER3, DLG2
279	5.55E-10	N	M phase	DYNC1H1, SMC1A, SMC2	SSSCA1,FZR1,PDS5B,PDS5A,CHEK1,SMC3,SMC4,NCAPD2,NCAPH,RAD21,NCAPG,STAG2,NEK6,NUDC,STAG1
8328	7.06E-09	Y	ionotropic glutamate receptor complex	GRIN1,GRIN2A,GRIN2B	GRIA1, GRIN3B, GRIA4
8066	2.31E-08	Y	glutamate receptor activity	GRM1, GRIN1,GRIN2A,GRIN2B	GRIA1, GABBR1, GRIN3B, GRIA4
7216	1.60E-04	Y	metabotropic glutamate receptor signaling pathway		HOMER3, HOMER1, HOMER2
50839	1.18E-02	N	cell adhesion molecule binding	GRIN2A,GRIN2B	PTPRT

Significant GO terms enriched in stage II network are given. If the ratio of the genes in the network for a given GO term to the total number of genes in that GO term is greater than 20%, ‘Y’ is indicated, otherwise ‘N’ is indicated. Hub genes identified in our analysis are indicated for each GO term.

### Analysis of progression to stage III

**Fig 4:**
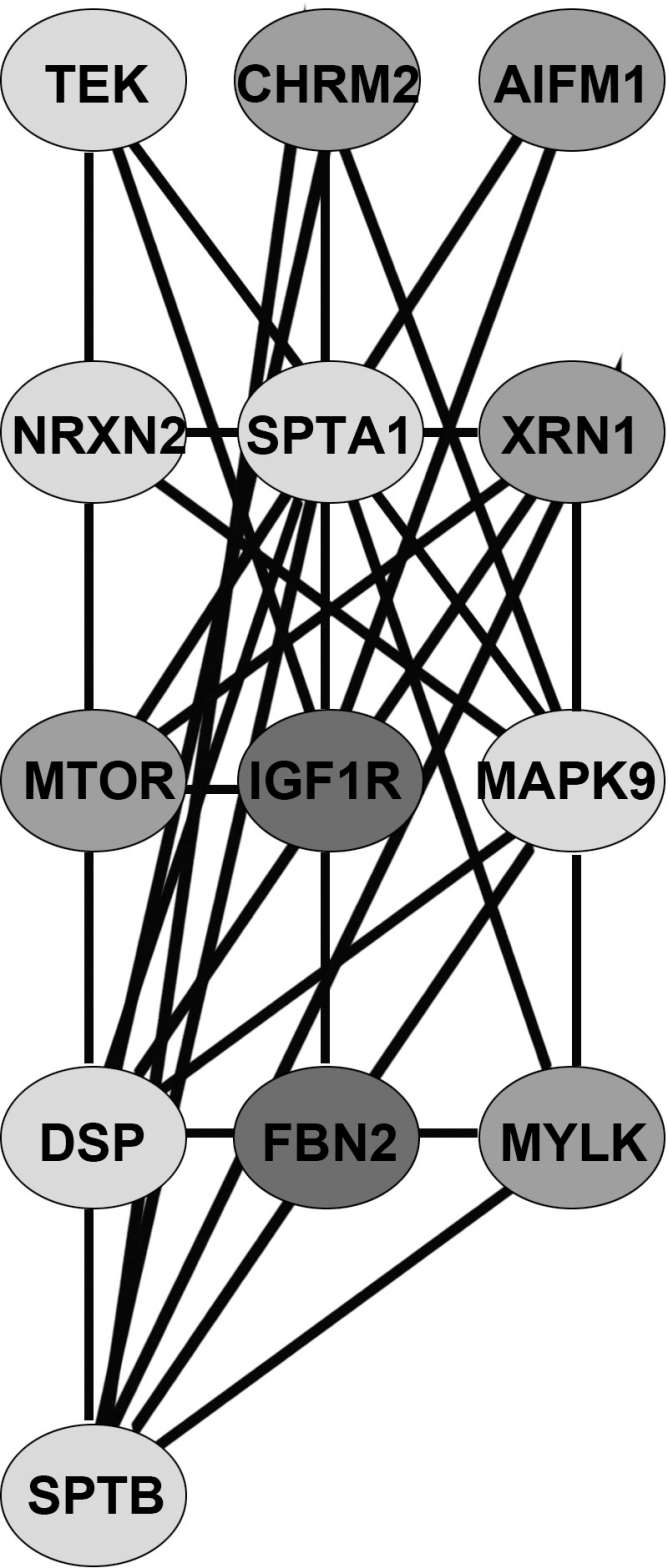
Network of cluster centres of stage-III network. Clustering was done using Moduland.

The reconstructed stage-III specific network (with node and edge attributes) is given in S2 File. Table 5 shows the central genes identified by centrality analysis for the stage III network. For progression to stage III, four hub driver genes were identified: IGF1R, CPS1, SPTA1, DSP. Six non-driver hub genes were identified: HEATR1, MAPK9, ARAF, PRKCE, PLEC, MSN. Table 6 shows the modules in the network identified by the ModuLand algorithm. All the four hub driver genes were classified as core members of the network clusters (Fig 4). The most significant enrichment in the GO analysis of the biological process was the regulation of actin cytoskeleton organization (q-value < 0.001; Table 7). The GO enrichment in the molecular function (“actin binding”) and cellular component (“actin cytoskeleton”, “spectrin”) provided further evidence for the involvement of SPTA1 and PLEC. DSP seemed to modulate the cell adhesion properties in advancing the cancer malignancy.

**Table 5:** Centrality analysis of stage-III network.

S.No	Betweenness centrality	Closeness centrality	Bottleneck centrality	Consensus Centrality (hubs)	Hub+driver genes
1	HEATR1	MACF1	ARAF	HEATR1	IGF1R
2	IGF1R	MAPK9	MAPK9	IGF1R	CPS1
3	MAPK9	IGF1R	PLEC	MAPK9	SPTA1
4	ARAF	SPTA1	MSN	ARAF	DSP
5	XRN1	PRKCE	HEATR1	PRKCE	
6	PRKCE	XRN1	IGF1R	CPS1	
7	CPS1	DSP	CPS1	PLEC	
8	PLEC	ARAF	SPTA1	MSN	
9	MSN	PLEC	UTP20	SPTA1	
10	MTOR	HEATR1	RICTOR	DSP	
11	MACF1	CPS1	MTOR		
12	UTP20	MSN	PIK3R3		
13	SPTA1	NRXN2	DSP		
14	DSP	COL17A1	COL17A1		
15	MAPK6	MAPK6	PRKCE		

The top 15 genes obtained by applying each centrality metric are given. The genes at the intersection of all the three metrics are designated as ‘hubs’. Genes that are both hub and driver are shown.

**Table 6:** Moduland decomposition of stage-III network.

S. No	Module centre	Eff. size of module	Driver gene members	Hub gene members
1	MTOR	15	RICTOR, UTP20, MAGEC1	HEATR1
2	SPTA1	11	SPTA1, MACF1	SPTA1, PLEC
3	DSP	12	DSP, ADAM28, PKD2L1	DSP, PRKCE
4	SPTB	7	MACF1	
5	NRXN2	8	NRXN3	
6	XRN1	16	XRN1, CPS1	CPS1
7	TEK	7	TEK	
8	MAPK9	14	MAPK6, ANK3	MAPK9, ARAF
9	IGF1R	7	IGF1R	IGF1R, MSN
10	FBN2	6	FBN2	
11	MYLK	7	MYLK	
12	CHRM2	4	CHRM2	
13	AIFM1	5	AIFM1	

Each module is represented by its centre and effective size. Hub genes identified by centrality analysis and driver genes are indicated by their module membership. Modules with the maximum number of hub genes could function as driver subnetworks in the pathogenesis of disease progression.

**Table 7:** GO enrichment analysis of stage-III network.

GO- ID	q-value	%size > 20	Description	Hub genes	Other genes in the network
7059	3.10E-12	N	chromosome segregation	SMC2, SMC1A	PDS5B,SMC3,SMC4,NCAPD2,NCAPH,RAD21,NDEL1,NCAPG,STAG2,NEK6,STAG1
7215	1.62E-10	Y	glutamate signaling pathway	GRIN1,GRIN2A,GRIN2B	HOMER3, GRIN3B,GRIA4,HOMER1, HOMER2
45202	1.62E-10	N	Synapse	GRIN1,GRIN2A,GRIN2B, GRM1, DLG4,CAMK2A	GABBR1,NLGN2,GRIN3B,GRIA4,CDH2,HOMER1, ADORA1,HOMER2,SLC17A7,SLC32A1, GRIA1,HOMER3, DLG2
279	5.55E-10	N	M phase	DYNC1H1, SMC1A, SMC2	SSSCA1,FZR1,PDS5B,PDS5A,CHEK1,SMC3,SMC4,NCAPD2,NCAPH,RAD21,NCAPG,STAG2,NEK6,NUDC,STAG1
8328	7.06E-09	Y	ionotropic glutamate receptor complex	GRIN1,GRIN2A,GRIN2B	GRIA1, GRIN3B, GRIA4
8066	2.31E-08	Y	glutamate receptor activity	GRM1,GRIN1,GRIN2A,GRIN2B	GRIA1, GABBR1, GRIN3B, GRIA4
7216	1.60E-04	Y	metabotropic glutamate receptor signaling pathway		HOMER3, HOMER1, HOMER2
50839	1.18E-02	N	cell adhesion molecule binding	GRIN2A,GRIN2B	PTPRT

Significant GO terms enriched in stage III network are given. If the ratio of the genes in the network for a given GO term to the total number of genes in that GO term is greater than 20%, ‘Y’ is indicated, otherwise ‘N’ is indicated. Hub genes identified in our analysis are indicated for each GO term.

### Analysis of progression to stage IV

**Fig 5:**
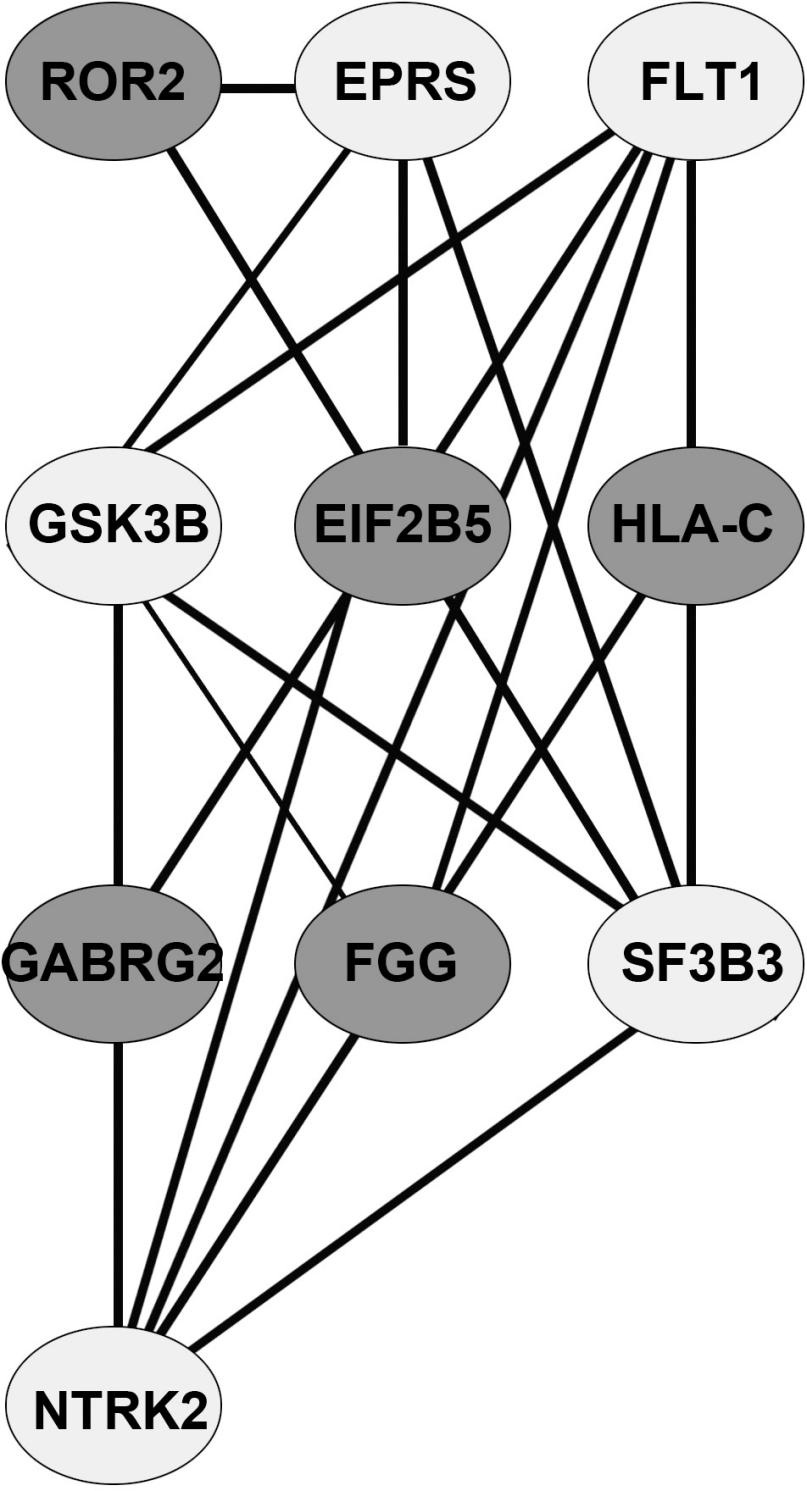
Network of cluster centres of stage-IV network. Clustering was done using Moduland.

The reconstructed stage-IV specific network (with node and edge attributes) is given in S3 File. Table 8 shows the central genes identified by centrality analysis for the stage IV network. For progression to stage IV, three hub driver genes were identified: GSK3B, GGT1, EIF2B5. Seven non-driver hub genes were identified: AKT1, PXN, SFN, GNAI2, CHKB, HSPA5, PLCG1. A ModuLand analysis of the network yielded a module centered at EIF2B5, which included all the three hub driver genes noted above (Table 9; Fig 5). A GO biological process enrichment analysis yielded ‘negative regulation of translational initiation in response to stress’ (q-value < 0.001; Table 10). This hit contained the EIF2B5 gene, which was also a member of the enriched cellular component ‘eukaryotic translation initiation factor 2B complex’ (q-value < 0.001). The GO molecular function enrichment analysis yielded the following: adenyl nucleotide binding containing hub genes GSK3B and HSPA5 (q-value = 0.001) and gamma-glutamyl transferase activity containing hub gene GGT1 (q-value ≈ 0.01). A connection with neurogenesis and oligodendrocyte development was seen (q-values < 0.001), which might seem surprising, but recent studies indicated a crucial link of these signaling pathways with the metastasis of colon cancer [26].

**Table 8:** Centrality analysis of stage-IV network.

S.No.	Betweenness centrality	Closeness centrality	Bottleneck centrality	Consensus Centrality (hubs)	Hub+driver genes
1	GSK3B	GSK3B	GSK3B	GSK3B	GSK3B
2	AKT1	PXN	AKT1	AKT1	GGT1
3	PXN	AKT1	HSPA5	PXN	EIF2B5
4	GGT1	VEGFA	CHKB	GGT1	
5	SFN	PLCG1	GGT1	SFN	
6	PPIF	EIF2B5	PXN	GNAI2	
7	GNAI2	CHKB	SFN	CHKB	
8	CHKB	GNAI2	PLCG1	HSPA5	
9	HSPA5	FLT1	EIF2B5	EIF2B5	
10	EIF2B5	HSPA5	QARS	PLCG1	
11	PLCG1	SFN	SSR4		
12	SF3B3	GGT1	GNAI2		
13	IARS	SF3B1	HECW1		
14	NTRK2	ITGAV	NTRK2		
15	MAGI1	ITGA9	SF3B3		

The top 15 genes obtained by applying each centrality metric are given. The genes at the intersection of all the three metrics are designated as ‘hubs’. Genes that are both hub and driver are shown.

**Table 9:** Moduland decomposition of stage-IV network.

S. No	Module centre	Eff. size of module	Driver gene members	Hub gene members
1	EIF2B5	14	EIF2B5	EIF2B5, CHKB, GGT1, GSK3B
2	FLT1	29	FLT1, TNC, ADCY8	GNAI2, PXN, PLCG
3	GSK3B	34	GSK3B, MAGI1, LCT	GSK3B, AKT1, HSPA5, SFN
4	NTRK2	7	NTRK2, SLAMF7	
5	EPRS	8	EPRS	
6	SF3B3	12	SF3B3, GGT1, SYT14	GGT1
7	HLA-C	5		
8	ROR2	4		
9	FGG	3	F13B	
10	GABRG2	4	GABRG2	

Each module is represented by its centre and effective size. Hub genes identified by centrality analysis and driver genes are indicated by their module membership. Modules with the maximum number of hub genes could function as driver subnetworks in the pathogenesis of disease progression.

**Table 10:** GO enrichment analysis of stage-IV network.

GO- ID	q-value	%size > 20	Description	Hub genes	Other genes in the network
5851	7.11E-09	Y	eukaryotic translation initiation factor 2B complex	EIF2B5	EIF2B2, EIF2B1,EIF2B, EIF2B4
22008	1.23E-05	N	Neurogenesis	EIF2B5, AKT1	WNT5A,NTF3,PTPRZ1,NF1,DLL1,FZD2,EIF2B1,BDNF,DYNLL2,TNR,VEGFA,ROR2,EIF2B2,EIF2B3,EIF2B4,DISC1
30817	3.76E-05	N	regulation of cAMP biosynthetic process	GNIA2	ADCY1,ADCY2,ADCY8,DRD5,ADCY5,NF1,NTRK2
30554	1.04E-03	N	adenyl nucleotide binding	GSK3B, HSPA5	ADCY1,FLT1,ADCY2,SGK3,MAGI1,TAOK1,ADCY8,ADCY5,CHKB,EPRS,QARS,CLPX,MARK1,IARS,AKT1,MUSK,CBWD1,RARS,NTRK2,ROR2,EIF2B2,DUS2L
43409	1.10E-03	N	negative regulation of MAPKKK cascade	AKT1	SPRY2, MAGI1,NF1
3840	1.27E-02	Y	gamma-glutamyl transferase activity	GGT1	GGT2

Significant GO terms enriched in stage IV network are given. If the ratio of the genes in the network for a given GO term to the total number of genes in that GO term is greater than 20%, ‘Y’ is indicated, otherwise ‘N’ is indicated. Hub genes identified in our analysis are indicated for each GO hit.

We performed the literature survey for these novel driver genes, to provide an insight into the possible roles of these genes in cancer progression. To our surprise, most of the genes were under studied in connection with colon cancer.

### Stage II hub genes

#### Dynein, Cytoplasmic 1, Heavy Chain 1(*DYNC1H1*)

DYNC1H1 has been shown to function in intracellular motility like protein sorting, movement of the organelles and dynamics of the spindles. A recent study has reported that *DYNC1H1* is mutated in ovarian cancer, pancreatic neuroendocrine neoplasms, and glioblastoma multiforme (GBM) [27]. In addition, the expression of *DYNC1H1* was significantly upregulated in three drug-resistant gastric cancer cell lines (5-fluorouracil (5FU), paclitaxel (TA) and cisplatin (DDP)-resistant gastric cancer cell lines) [28].

#### Glutamate Receptor, Ionotropic, N-methyl D-aspartate 2A (*GRIN2A*)

GRIN2A is a member of the glutamate-gated ion channel proteins. It is a subunit of N-methyl-D-aspartate receptor. The activation of these receptors will increase the influx of calcium resulting in the triggering of several signaling cascades. In 2011, Wei et al., has identified that *GRIN2A* was mutated in 33% of melanoma samples [29]. Furthermore, D’mello et al., has provided evidence that the mutations in this gene are correlative to the progression of melanoma[30].

#### Glutamate receptor, metabotropic 1 (*GRM1*)

GRM1 was shown to activate phospholipase C. GRM1 was associated with various diseases such as depression and cancer. GRM1 has been implicated in prostate cancer [31], following identification of novel mutations and single nucleotide polymorphisms. In addition, *GRM1* was over expressed in melanoma and ectopic overexpression of this gene in melanocytes resulted in neoplastic transformation [32]. Finally, studies with breast epithelial cells demonstrated that GRM1 cooperates with other factors in the hyperplastic mammary epithelium resulting in progression of breast cancer [33].

#### Discs, Large Homolog 4 (*DLG4*)

*DLG4*(also called as postsynaptic density protein 95) has shown to be expressed in normal cervical keratinocytes. The expression of *DLG4* is significantly decreased in cervical cancer cell lines. Also, the tumorigenicity of CaSki cells was repressed following overexpression of *DLG4*[34]. In addition, Hering et al., has shown that DLG4 cooperates with Frizzled proteins to regulate the WNT signaling pathway, which has been implicated in multiple types of cancer progression, including colon cancer [35]. Table 3 shows that DLG4 is the module center of a key module of the stage II network.

#### Structural maintenance of chromosomes 2 (*SMC2*)

SMC2 plays a role in chromosomal stability. It is a subunit of condensing protein complexes that are shown to be involved in the condensation of the chromosomes. Table 3 shows that SMC2 is located at the center of a module of the stage II network. Je et al., has reported that *SMC2* is mutated in gastric and colon cancer tissues suggesting its involvement in cancer progression [36]. Interestingly, beta-Catenin a key molecule in colorectal cancer has been shown to directly bind and regulate the transcription of *SMC2*[37].

#### Phospholipase C gamma 1 (*PLCG1*)

PLCG1 has been shown to play a significant role in intracellular signaling pathways as well as an increased apoptotic resistance and invasiveness of the cells [38]. PLCG1 levels were significantly increased in breast cancer tissues compared to normal [39]. Raimondi et al., has demonstrated a link between PLCG1 and phosphoinositide-dependent kinase 1 (PDK1), and their significance in the process of cancer cell invasion[40]. In addition, Park et al., has reported that PLCG1 levels are significantly high in adenomas and carcinomas compared to the normal colonic mucosa suggesting its role in progression of colon cancer[41].

#### Glutamate receptor, ionotropic, N-methyl D-aspartate 2B (*GRIN2B*)

GRIN2B is a glutamate-gated ion channel with very high calcium permeability. Park et al., has shown that the *GRIN2B* promoter region is hypermethylated during breast cancer progression[42]. Similar epigenetic changes at the *GRIN2B* locus could be involved in driving the progression of colon cancer.

#### Ras Homolog Family Member G (*RHOG*)

RHOG is a member of Rho family of GTPases. RHOG is lesser characterized among Rho family members, and its role in cancer progression is unknown. It has been shown to regulate morphological changes in cells. Also, RHOG has been demonstrated to promote cell survival through activation of PI3Kinase and Akt[43]. *RHOG* has been shown to be upregulated in glioblastoma compared to a non-neoplastic brain. In addition, it has been shown to mediate glioblastoma cell invasion following cMet and EGFR stimulation [44].

#### Structural maintenance of chromosomes 1A (*SMC1A*)

SMC protein family contains 6 members from SMC1 to SMC6 with varying functions. *SMC1A* is mutated in various malignant carcinomas. Down regulation of *SMC1A* resulted in growth inhibition in lung adenocarcinoma cells [45]. Furthermore, knockdown of *SMC1A* significantly suppressed the proliferation of the glioblastoma cells [46]. Recently, Wang et al., has published that SMC1A is a predictive factor for poor prognosis of colon cancer[47].

#### Glutamate Receptor Ionotropic, NMDA 1 (*GRIN1*)

GRIN1 is the under studied member of the glutamate receptor family with regards to cancer. There is only one study showing the interaction of GRIN1 and GRIN2A and their role in anchorage independent growth of melanoma cells[48].

#### Calcium/Calmodulin-Dependent Protein Kinase II Alpha (*CAMK2A*)

CAMK2A is a serine/threonine protein kinase. Yuan et al., has demonstrated that CaMKII played a very important role in osteosarcoma proliferation, and this could be a therapeutic target for osteosarcoma[49].

### Stage III hub genes

#### Insulin-like Growth Factor 1 Receptor (*IGF1R*)

This tyrosine kinase receptor plays a critical role in cellular transformation events following binding of insulin-like growth factor. It is overexpressed in various malignant tissues, and it enhances cancer cell survival by inhibiting apoptotic cell death. In addition, there was a significant correlation between the expression of *IGF1R* with colorectal tumor size and depth of tumor invasion [50]. Finally, Kucab et al., has clearly highlighted the role of IGF1R in breast cancer metastasis[51].

#### Carbamoyl phosphate synthetase 1 (*CPS1*)

CPS1 is a mitochondrial enzyme involved in the urea cycle. Recent studies have mentioned that the expression of CPS1 is a negative prognostic factor in rectal cancers that receive concurrent chemoradiotherapy[52]. In addition, Li et al., has demonstrated the utilization of the anti-CSP1 for the detection of circulating tumor cells in hepatocellular carcinoma (HCC)[53]. Finally, studies have also shown that *CPS1* expression in human HCC cells is silenced by DNA methylation [54].CSP1 could be a potential biomarker for HCC.Milinkovic et al., has identified CPS1 as genetically altered in malignant glioma patient samples [55].

#### Spectrin, alpha, erythrocytic 1 (SPTA1)

SPTA1 is a scaffold protein that functions in determining cell shape and organization of organelles. These families of proteins are primarily composed of spectrin repeats involved in dimer formation. Recent studies have shown that β2-spectrin is implicated in colorectal and pancreatic cancer, where it regulates the transcriptional activators SMAD to affect transforming growth factor beta (TGFβ) signaling pathway. Dysregulation of TGFβ signaling through loss of β2-spectrin inappropriately activates Wnt signaling and promotes tumorigenesis [56]-[57]. In addition, spectrin family of proteins has been shown to play a role in hepatocellular cancer through regulation of cyclin D1 [58]. Interestingly, they have been shown to contribute to drug resistance in ovarian cancer [59]. Furthermore, the increase in the expression and heterogeneity of the cytoplasmic spectrin is associated with the invasiveness of malignant melanoma and squamous-cell carcinoma [60].

#### Desmoplakin (*DSP*)

DSP is an essential component of intercellular junctions called desmosomes. Loss of DSP expression has been shown to play an important role in breast cancer progression and metastasis [61]. Papagerakis et al., has demonstrated the utility of DSP as a marker for evaluating the risk of oropharyngeal cancer metastasis[62].

#### HEAT Repeat Containing 1 (*HEATR1*)

HEATR1 is known to be to be involved in the biogenesis of ribosomes. Liu et al., has recently demonstrated that HEATR1 plays a significant role in pancreatic cancer cell drug resistance, and that HEATR1 regulates Akt pathway[63]. In addition, Wu et al., has demonstrated that the overexpression of *HEATR1* in glioblastoma cells resulted in the induction of a cytotoxic T lymphocyte response to its epitopes, which allowed for the selective targeting of the glioblastoma cells and glioma stem like cells[64].

#### Mitogen-activated protein kinase 9 (*MAPK9*)

MAPK9 also called as c-Jun N-terminal kinases (JNK2), belongs to the family of MAPK kinases, and has shown to regulate multiple cellular processes including proliferation, differentiation, and transcription regulation. Ahmed et al., has demonstrated that JNK2 mediated suppression of JNK1 apoptotic pathway is required for the survival of cancer cells[65]. In addition, blocking the expression of JNK2 has significantly inhibited the migration ability of breast cancer cells [66].

#### A-Raf serine/threonine kinase (*ARAF*)

*ARAF* proto-oncogene is a member of RAF subfamily, and has been implicated in cell growth and development. Mutations in this gene have been shown to transform immortalized human airway epithelial cells [67]. In addition, Mooz et al., has shown that ARAF has an essential part in stimulating MAPK activity and cell migration[68].

#### Protein Kinase C, Epsilon (*PRKCE*)

PRKCE is another serine- and threonine-specific protein kinase activated by calcium and diacylglycerol. PRKCE is correlated with cell transformation and tumorigenesis. It has been shown to suppress apoptotic death of cells. The oncogenic potential of PRKEC in thyroid cancer was demonstrated by Zhang et al [69]. PRKCE has been shown to play an important role in promoting an aggressive metastatic breast cancer phenotype [70].

#### Plectin (*PLEC*)

PLEC is the cytolinker protein shown to regulate the tissue integrity, actin organization and cell migration. Yoneyama et al., has demonstrated the role of plectin in facilitating cancer cell invasion and metastasis[71]. In addition, plectin has been shown to regulate invasiveness by modulating actin assembly in SW480 colon cancer cells [72].

#### Moesin (*MSN*)

MSN is a membrane-organizing extensions spike protein. Moesin is identified to be present in the filopodia and other membranous projections that play a critical role in cell movement and cell signaling. A recent study has demonstrated that microRNA 200b inhibited breast cancer metastasis through regulation of moesin expression. In addition, increased expression of moesin has been shown to have an association with poor relapse-free survival [73]. Interestingly, phosphorylation of moesin by G protein-coupled receptor kinase has been shown to regulate prostate cancer metastasis [74].

### Stage IV hub genes

#### Glycogen synthase kinase-3 (*GSK3B*)

GSK-3 is a serine-threonine kinase and shown to play a role in a wide range of cellular processes. Inhibition of GSK3B activity by Akt has been shown to influence the cancer progression. Furthermore, studies have shown that inhibition of GSK3 induced invasiveness of the breast cancer. Wnt signaling plays a critical role in colon cancer progression, and GSK3B is known to regulate this pathway[75]. Understanding the mutations in *GSK3B* will lead to improved therapies for colon cancer.

#### Gamma-Glutamyl transferase 1 (*GGT1*)

GGT1 is a membrane-bound enzyme catabolizing reduced glutathione to cysteine and glycine. GGT is a marker of oxidative stress in cells. Increased expression of *GGT* has shown to elevate the risk of progression of cervical cancer [76]. In addition, elevated levels of GGT are shown to be associated with increased invasion of melanoma cells in both in vitro and in vivo studies [77].

#### Eukaryotic Translation Initiation Factor 2B, Subunit 5 Epsilon (*EIF2B5*)

EIF2B5 is a regulator of protein synthesis. It has been associated with ovarian cancer, and angiogenesis[78]. In addition, genome-wide array study has identified EIF2B5 gene copy alteration in esophageal squamous-cell carcinoma patients [79]-[80].

#### Paxillin (*PXN*)

PXN is a cytoskeleton protein shown to be involved in cell adhesion. *PXN* mutations are associated with lung adenocarcinoma and are an independent predictor of survival and relapse of non-small cell lung cancer[81]. In addition, there are studies showing the role of PXN in the metastasis of osteosarcoma and prostate cancer [82,83]. A very recent study reported that PXN regulates tumor invasion, and is responsible for poor patient outcome in colorectal cancer patients [84].

#### Stratifin (*SFN*)

SFN is an adapter protein shown to regulate several signaling pathways. The promoter regions of *SFN* in most of the invasive lung adenocarcinoma samples are methylated to silence *SFN* expression[85]. In 2015, Shiba et al demonstrated the role of SFN on lung tumor development and progression[86].

#### Guanine Nucleotide Binding Protein, Alpha Inhibiting Activity Polypeptide 2 (*GNAI2*)

GNAI2 has shown to be involved as modulators or transducers in several transmembrane signaling pathways. GNAI2 has been implicated in ovarian cancer, where it acts as a driver of cancer progression[87]. Jiang et al., has reported the proto-oncogenic role of GNAI2 in tongue squamous-cell carcinoma initiation and progression[88].

#### Choline Kinase Beta (*CHKB*)

CHKB has been shown to be involved in the biosynthesis of phospholipids. There is very little information on the role of CHKB on cancer. However, TP53 and CHKB may regulate CDK4/6 collaboratively to suppress the progression of ovarian cancer[89]. Gallego-Ortega et al., has attempted to determine the involvement of CHKA and CHKB in cancer[90].

#### Heat Shock 70kDa Protein 5 (*HSPA5*)

HSPA5 is involved in the folding and assembly of proteins. It has been shown to regulate the anti-apoptotic unfolded protein response signaling network, which provides a ready mechanism for promoting cancer progression and metastasis [91]. Recent study by Chang et al., has demonstrated the role of HSPA5 on breast cancer cell migration and invasion[92]. Interestingly, Booth et al., 2014, has reported that OSU-03012, a cyclo-oxygenase inhibitor, targets HSPA5 and induces cancer cell death[93].

#### AKT1

AKT1 is a well-known driver of cancer [94]-[95], hence its appearance in our analysis might seem surprising. Despite the elimination of AKT1 at the screening stage, repopulating the network with interacting partners seemed to have led to the emergence of AKT1 in the network. In fact, it was the only entry in the Cancer Gene Census that re-appeared as a hub. This could be interpreted as additional evidence of the essential role of AKT1 in the metastasis of cancer.

In summary, for each stage of colon cancer, we have identified consensus driver genes, consensus hub genes, and the genes at their intersection referred as ‘hub driver’ genes. Each of these genes is a potential novel diagnostic biomarker of the stage of colorectal cancer. We have discussed the probable association of each biomarker with the progression of cancer, and in each case, we have found ample evidence that the gene in question constituted a missing link in the current understanding of the progression of colorectal cancer. It would appear that some of these biomarkers might even be involved in the progression of multiple unrelated cancers. Each identified biomarker represents a potential target for chemotherapeutic intervention in colorectal cancer. The modularity analysis reaffirmed these key genes, identifying the larger subnetworks of which they are a part. More than individual genes, it could be the subnetworks that are critical to the cancer progression and hence these subnetworks could serve as diagnostic biomarkers for the stage of colorectal cancer as well as provide targets for therapy. The Gene Ontology analysis has shed light on certain novel mechanisms that might underlie the progression of colorectal cancer to its next malignant stage. Our study design has been effective in revealing novel biomarkers and mechanisms that are driving each stage of cancer.

## Conclusion

Our study has yielded many novel stage-specific biomarkers which would boost current strategies towards critical early-stage diagnosis of the stage of colorectal cancer as well as target selection for rational and personalized cancer treatment. We have identified *DYNC1H1*,*GRIN2A*, and *GRM1* as novel hub driver genes for the stage-II progression of colon adenocarcinoma. *IGF1R*, *CPS1*, *SPTA1* and *DSP* were identified as novel hub driver genes for the stage-III progression, and *GSK3B*, *GGT1* and *EIF2B5* were identified as novel hub driver genes for stage-IV progression. Prognosis is clearly inversely correlated with the stage of cancer and hence the biomarkers discussed above could predict the prognosis based on the stage-specificity. Our results provide multiple concrete directions for the deeper investigation of the biology of colorectal cancer malignancy in the future. Our methodology is extendable to the analysis of multiple types of cancer progression to yield novel useful biomarkers.

## Supporting Information

S1 File Stage-II network with centrality metrics. (XGMML)

S2 File Stage-III network with centrality metrics. (XGMML)

S3 File Stage-IV network with centrality metrics. (XGMML)

S1 Table Stagewise novel driver genes used in network construction. (DOCX)

S2 Table Power-law behavior of the node distribution of all the stage-specific networks. (DOCX)

S3 Table Summary of topological parameters of all the stage-specific networks. (DOCX)
