# Supplementary material for: Computational Identification of Novel Stage-Specific Biomarkers in Colorectal Cancer Progression
Source: PLoS One. 2016 May 31;11(5):e0156665. doi: 10.1371/journal.pone.0156665 (PMC4887059; doi:10.1371/journal.pone.0156665)
Supplement: S1 Table — (DOCX) [file pone.0156665.s004.docx]

| **S.No.** | **Gene set for progression to stage II** | **Gene set for progression to stage III** | **Gene set for progression to stage IV** |
| --- | --- | --- | --- |
| 1 | ARHGAP5 | ABI3BP | ADCY2 |
| 2 | CLSPN | ADAM28 | ADCY8 |
| 3 | DEFB126 | AIFM1 | ASPM |
| 4 | DYNC1H1 | ANK3 | CDH18 |
| 5 | FLG | CHRM2 | CDH9 |
| 6 | FLT1 | CPS1 | DYNC2H1 |
| 7 | GRIA4 | CYSLTR2 | EIF2B5 |
| 8 | GRIN2A | DSP | EPRS |
| 9 | GRM1 | FBN2 | F13B |
| 10 | IL1RAPL1 | GRID1 | FLT1 |
| 11 | IQGAP2 | HLA-DQA1 | GABRG2 |
| 12 | KCNQ5 | IGF1R | GGT1 |
| 13 | KRTAP4-1 | LPA | GPRIN2 |
| 14 | LRRC7 | MACF1 | GSK3B |
| 15 | MKI67 | MAGEC1 | HECW1 |
| 16 | MYH2 | MAP1B | KIR2DL3 |
| 17 | NCAPD2 | MAPK6 | LCT |
| 18 | PAPPA | MYLK | MAGI1 |
| 19 | PCDH17 | NCOA6 | MARK1 |
| 20 | PEAK1 | NLGN4X | NTRK2 |
| 21 | PPP1R12B | NRXN3 | PTPRS |
| 22 | PTPRT | PKD2L1 | PTPRZ1 |
| 23 | RBM38 | RICTOR | ROR2 |
| 24 | SOX9 | SLCO1B1 | SF3B3 |
| 25 | STAG1 | SORCS1 | SLAMF7 |
| 26 | UBE4B | SPTA1 | SYNE1 |
| 27 | VCAN | TEK | SYT14 |
| 28 |  | UTP20 | TNC |
| 29 |  | XRN1 | UQCRC2 |
| 30 |  | ZC3H13 | ZMYND8 |
| 31 |  | ZNF804A |  |
