# Supplementary material for: Computational Identification of Novel Stage-Specific Biomarkers in Colorectal Cancer Progression
Source: PLoS One. 2016 May 31;11(5):e0156665. doi: 10.1371/journal.pone.0156665 (PMC4887059; doi:10.1371/journal.pone.0156665)
Supplement: S2 Table — (DOCX) [file pone.0156665.s005.docx]

| **Stage** | **Power-law distribution** | **Correlation coefficient of power-law fit (R)** | **R-squared of power-law fit**^*^ | **R-squared of best Line- fit** |
| --- | --- | --- | --- | --- |
| II | y ≈ 25 x **^-0.895^** | 0.884 | 0.723 | 0.457 |
| III | y ≈ 42 x **^-1.197^** | 0.929 | 0.817 | 0.455 |
| IV | y ≈ 39 x **^-1.133^** | 0.943 | 0.781 | 0.450 |

^*^R-Squared is computed on logarithmized values.
