# Supplementary material for: Computational Identification of Novel Stage-Specific Biomarkers in Colorectal Cancer Progression
Source: PLoS One. 2016 May 31;11(5):e0156665. doi: 10.1371/journal.pone.0156665 (PMC4887059; doi:10.1371/journal.pone.0156665)
Supplement: S3 Table — (DOCX) [file pone.0156665.s006.docx]

| **№** | **Network parameter** | **Stage II** | **Stage III** | **Stage IV** |
| --- | --- | --- | --- | --- |
| 1 | Number of connected components | 1 | 1 | 1 |
| 2 | Number of nodes | 109 | 109 | 115 |
| 3 | Number of edges | 396 | 199 | 297 |
| 4 | Clustering coefficient | 0.317 | 0.137 | 0.253 |
| 5 | Network density | 0.042 | 0.023 | 0.028 |
| 6 | Network diameter | 9 | 14 | 12 |
| 7 | Network radius | 5 | 8 | 6 |
| 8 | Network heterogeneity | 0.869 | 0.748 | 0.805 |
| 9 | Network centralization | 0.155 | 0.071 | 0.132 |
| 10 | Any self-loops? | No | No | No |
| 11 | Characteristic path length | 3.942 | 6.932 | 4.852 |
| 12 | Avg. number of neighbors | 4.550 | 2.440 | 3.217 |
| 13 | Shortest paths | 100% | 100% | 100% |
